# Understanding Nash epidemics

**DOI:** 10.1073/pnas.2409362122

**Published:** 2025-02-27

**Authors:** Simon K. Schnyder, John J. Molina, Ryoichi Yamamoto, Matthew S. Turner

**Affiliations:** ^a^Institute of Industrial Science, The University of Tokyo, Tokyo 153-8505, Japan; ^b^Department of Chemical Engineering, Kyoto University, Kyoto 615-8510, Japan; ^c^Department of Physics, University of Warwick, Coventry CV4 7AL, United Kingdom; ^d^Institute for Global Pandemic Planning, University of Warwick, Coventry CV4 7AL, United Kingdom

**Keywords:** epidemiology, control theory, mean-field games, game theory, mathematical modeling

## Abstract

Faced with a dangerous epidemic humans will spontaneously social distance to reduce their risk of infection at a socioeconomic cost. Compartmentalized epidemic models have been extended to include this endogenous decision making: Individuals choose their behavior to optimize a utility function, self-consistently giving rise to population behavior. Here, we study the properties of the resulting Nash equilibria, in which no member of the population can gain an advantage by unilaterally adopting different behavior. We leverage an analytic solution that yields fully time-dependent rational population behavior to obtain, 1) a simple relationship between rational social distancing behavior and the current number of infections; 2) scaling results for how the infection peak and number of total cases depend on the cost of contracting the disease; 3) characteristic infection costs that divide regimes of strong and weak behavioral response; 4) a closed form expression for the value of the utility. We discuss how these analytic results provide a deep and intuitive understanding of the disease dynamics, useful for both individuals and policymakers. In particular, the relationship between social distancing and infections represents a heuristic that could be communicated to the population to encourage, or “bootstrap,” rational behavior.

## Significance

Social behavior during epidemics is a collective phenomenon: individuals adjust their activity depending on the epidemic state which itself is generated by that same behavior. Game theoretic analysis shows that such dynamics can give rise to a Nash equilibrium. Previously, our analytic understanding of Nash equilibria in epidemics has been extremely limited, leaving us reliant on numerical solutions. Here, we identify an exact analytic expression for fully time-varying Nash equilibrium behavior and resultant disease dynamics. In particular, the strength of social distancing is proven to be proportional to both the perceived infection cost and prevalence. Remarkably, this gives a posteriori justification for the sort of simple heuristics developed to understand diseases like HIV.

Throughout history, epidemics caused by infectious diseases have caused considerable harm to humans. Early models of the epidemic spread of infectious disease (1) treated the behavior of the population as unaffected by the threat of the disease, and thus constant. More recent studies sometimes assume individuals are able to adjust their behavior in reaction to the threat of an epidemic (2–17). At first, the behavioral changes exhibited by populations during an epidemic were modeled ad hoc, with behaviors determined by some arbitrary relationship, e.g., a function of the current disease prevalence (2, 4, 6, 8). Later, economics-based approaches were developed in which individuals were able to weigh the costs and benefits of outcomes in order to make choices about their behavior. Crucially, this behavior can be highly dynamic and time-dependent: they can reduce their social activity when infections are high, in order to reduce the probability of becoming infected themselves, provided that the avoided health costs outweigh the social and economic costs caused by the reduction of their activity.

A common assumption is that individual agents act rationally, i.e., to maximize an objective function or economic utility (3). This remains one of the fundamental assumptions of modern economic theory despite its limitations (18). Rational individuals, who aim to maximize their individual objective function, would choose to target a Nash equilibrium (3, 5, 7, 14, 19, 20) rather than the global utility maximum, which instead requires a coordinated effort to maximize a collective objective function (12, 21–23). Although not the focus of the present work we note that it is possible to bring a Nash equilibrium into alignment with the global optimum (3, 21, 24–26 and the chapter by Mark Gersovitz in ref. 4), e.g., via tax and subsidy incentives (27) which can be designed to bias rational individual behavior appropriately. Even with government intervention, it is however challenging to target complete eradication of a disease (see ref. 3 and the chapter by Mark Gersovitz in ref. 4).

Here, we formulate a simple compartmental SIR disease dynamics in which the fully time-dependent social activity of the population affects the disease transmission rate. The population behavior arises from the choice of behavior made by individuals. We analyze this decision making problem as a mean-field game for a representative individual reacting to a population behavior, which afterward is made self-consistent with the behavior of the individual. This approach requires us to define the individual’s utility: we focus on the case where the cost of infection is constant and where the government takes no role in directing the response to the epidemic. This situation has been already discussed, e.g., refs. 5, 12, and 19 among many others, but only using numerical solutions. We assume that the cost of social distancing is quadratic in the strength of behavioral modification and that it is paid by all compartments. This is akin to assuming largely asymptomatic but costly infections. Fortuitously, this allows us to exactly calculate the fully time-dependent social distancing behavior during an epidemic corresponding to the Nash equilibrium of such a mean-field game. Since it is extremely challenging to eradicate infectious diseases, we focus here on scenarios that ultimately lead to herd immunity.

Compartmental models like ours can be extended to more accurately represent the complexity of epidemics and the systems in which they occur, such as birth and death dynamics, additional compartment types with different risk and behavior profiles (19, 28–34), seasonal effects (35), waning immunity (34, 36), e.g., due to new variants (37), the dynamics of information, such as word-of-mouth propagation or the imitation of behavior (4), as well as spatial, transmission, or behavioral heterogeneity (4, 30, 38–42). Other approaches feature spatial (43) and temporal networks (44, 45), and/or agent-based models (46–49). Others have worked to incorporate uncertainty and noise, by considering stochastic control (50–54), decision making under uncertainty (55, 56) and by understanding the robustness of control (57–59). Model structure, epidemiological properties, or the effectiveness of interventions can be inferred from observed data (60–64). We do not consider any of the above here, nor policy interventions such as vaccination and treatment strategies (3, 4, 6, 8, 9, 14, 16, 22, 34, 47, 49, 65–73), or isolation, testing, and active case-tracing strategies (74–77). We also ignore the situation where a vaccine becomes available during the epidemic. While the early arrival of a vaccine would have consequences for both equilibrium and globally optimal behavior (5, 12, 20, 78), this lies outside of the scope of this work. Finally, we also remark on the intriguing possibility of allowing individual opinions to directly influence policy makers (79). Even the most sophisticated models struggle to make quantitative predictions during epidemics. The strength of simple compartmental models like ours is that while they are highly idealized, they provide a deep and intuitive qualitative understanding.

Nash equilibria are widely believed to occur within such idealized models that incorporate endogenous behavior during epidemics. However, until this work, solutions have only been accessible numerically. This is because the problem is intrinsically nonlinear, both at the level of the epidemiological dynamics and the objective function, leading to nonlinear control equations. Here, we provide an analytic solution to the nonlinear time-dependent equilibrium control equations for social distancing during an epidemic. This also demonstrates the existence of such a Nash equilibrium. In the limit of vanishing infection cost our results trivially recover the known analytic solutions for compartment models with constant basic reproduction number (1, 80–83), i.e., without endogenous rational behavior. We believe that Nash equilibria of idealized epidemic models provide a point of reference for understanding self-organized, self-interested behavior during an epidemic.

## Epidemic Dynamics

We use a standard SIR compartmentalized model (1) for the epidemic. The population is divided into susceptible, infected and recovered compartments, the latter implicitly including fatalities. The compartments evolve over time as

[1] $$\begin{array}{cc} \frac{d}{\mathrm{dt}}s & =-k\;s\;i\\ \frac{d}{\mathrm{dt}}i & =k\;s\;i-i\\ \frac{d}{\mathrm{dt}}r & =i, \end{array}$$

The time dependence of s(t), i(t), r(t) and k(t) is omitted for brevity. We normalize the compartments, 1=s+i+r. The model contains a single timescale which expresses the duration of an infection, and we have rescaled the equations so that time t is measured in units of this single timescale. The initial conditions are set as $s\left(0\right)=s_{0}$, $i\left(0\right)=i_{0}$, $r\left(0\right)=r_{0}$, with $s_{0},i_{0},r_{0}\geq 0$ and $s_{0}+i_{0}+r_{0}=1$. In all figures, we arbitrarily select a time origin t=0, where the epidemic is in its very early stages according to $r_{0}=i_{0}/\left(R_{0}-1\right)=10^{-6}$ with $s_{0}=1-i_{0}-r_{0}$.

The population’s average social activity behavior is encoded in the current transmission rate, assumed to satisfy k(t)≥0 although our analytic results later suggest a stronger bound k(t)≥1. We assume that the disease exhibits a natural level of activity in the absence of any behavioral modification that is a constant known as the basic reproduction number $R_{0}$. Below we use the case $k\left(t\right)=R_{0}$ to establish a nonbehavioral baseline dynamics for comparison.

## Nash Equilibrium Behavior

In order to study self-organized behavior, we imagine an average individual making decisions about their own behavior. This represents a mean-field game (84, 85), for the Nash equilibrium of which a set of ordinary differential equations can be straightforwardly derived (14, 86).

The individual’s effect on the epidemic is negligible but they can influence their own fate by selecting a strategy κ(t)≥0 which it is initially assumed can differ from the population-averaged strategy k(t). The probabilities that an individual is in each of the compartments evolve over time according to

[2] $$\begin{array}{cc} \frac{d}{\mathrm{dt}}\psi_{s} & =-\kappa \psi_{s}i\\ \frac{d}{\mathrm{dt}}\psi_{i} & =\kappa \psi_{s}i-\psi_{i}, \end{array}$$

Lowering κ(t) directly increases the probability of the individual remaining susceptible and reduces their probability of becoming infectious. While these equations are similar to Eq. **1**, they express the fact that the individual, while susceptible, can only be infected by the population (with fraction of infected i) and not by itself.

We assume that an individual has rational interests that can be captured by an objective function or utility. In general this will depend on both their own and the population behaviors, U(κ(t),k(t)). The individual seeks to maximize this objective function. Assuming that the population consists of identical individuals, a Nash equilibrium exists if there is a strategy k=κ(t), adopted by the population, and the individual cannot improve their outcome by unilaterally deviating from the behavior κ,

[3] $$U\left(\tilde{\kappa }\left(t\right),\kappa \left(t\right)\right)\leq U\left(\kappa \left(t\right),\kappa \left(t\right)\right),\;\;\text{for any}\;\tilde{\kappa }\left(t\right).$$

In order to find this Nash strategy one first maximizes U(κ,k) over κ for an arbitrary, exogenous k (14). This constitutes a standard constrained optimization problem. To make the strategy self-consistent, one then assumes that all individuals in the population would optimize their behavior in the same way, and therefore k=κ. This then automatically results in $\psi_{s}=s$, $\psi_{i}=i$ with dynamics that corresponds to the Nash equilibrium.

In this work, we focus on an idealized individual objective function or utility U with; see ref. 26,

[4] $$\begin{array}{cc} U= & \;\int_{0}^{t_{f}}u\left(t\right)dt+U_{f}, \end{array}$$

[5] $$u=\;-\alpha \psi_{i}-\xi {\left(\kappa -R_{0}\right)}^{2}.$$

The integral is truncated at a final time $t_{f}$ (more on this below). We neglect economic discounting of events at later times. The average infection cost is given by α (this also includes the cost of death) with α≥0. The social and financial costs of social distancing are parameterized by a constant ξ>0. In what follows, we choose to work in units of utility in which ξ=1, without loss of generality. The quadratic form of this social distancing term encodes that it is costly to deviate from one’s default behavior and ensures that an individual would naturally select behavior corresponding to $\kappa =R_{0}$ if there were no epidemic (or infection bore no cost). We make the common choice of truncating the utility integral at a final time $t_{f}$ at which a perfect vaccine becomes available, e.g., see refs. 5, 12, 29, and 78. Introducing such a final time helps in obtaining a well-stated boundary value problem for the analytic solution. We assume that at that time the susceptible compartment becomes completely and perfectly vaccinated, i.e., the fraction of susceptibles instantaneously drops to 0 at $t=t_{f}$. However, since infections are not cured by a vaccination, the fraction of the population that is infectious at vaccination time still has to recover, which happens exponentially over time. In order to include this contribution to the utility we integrate the infection cost accumulated for $t>t_{f}$ into the term $U_{f}=-\alpha \;\psi_{i}\left(t_{f}\right)$, see *Materials and Methods* for a brief calculation.

If the vaccination time $t_{f}$ is very large, then the population reaches herd immunity before any vaccine arrives and $i(t_{f})\to 0$. We focus on this situation, for which we analytically calculate the social distancing behavior and resulting epidemic dynamics. If $t_{f}$ is small enough it can have a qualitative effect on rational decision-making (5, 12, 78): for any given time, social distancing tends to be stronger the sooner a vaccination is expected to arrive. Simply speaking, the smaller $t_{f}$ is, the smaller is the maximum cost of suppressing the disease over the duration of the epidemic. A rough criterion for when the vaccination time becomes relevant for behavioral modification is thus given when the maximum cost of suppressing the disease over the duration of the epidemic becomes comparable to the infection cost, $\alpha ∼R_{0}^{2}t_{f}$ for our utility. Therefore, one would expect social distancing to become nonnegligibly affected by the vaccination event when $t_{f}<\alpha /\left(R_{0}^{2}\right)$. However, this scenario lies outside of the scope of this work as it severely complicates the analysis. Therefore, we choose $t_{f}$ finite but large enough such that $i(t_{f})\to 0$ holds self-consistently. It is an open question whether the analytic solution provided here can be generalized for short times $t_{f}$.

Since the utility function is convex, we expect that the optimization problem has a (unique) solution. We directly demonstrate uniqueness and existence by calculating the analytic solution to this problem below.

### Discussion of the Utility Function and Comparison to Other Work.

Our approach is most similar to refs. 5, 12, 19, 26, 78, and 87. In the following, we discuss the main similarities and differences of our work to theirs.

Our assumption that the infection cost α is constant is a very common one, e.g., refs. 5, 12, 78, and 87. In contrast, there are examples where this is not a good assumption, e.g., when hospital capacity is limited the infection cost can depend on the prevalence of infections in the population. Including this effect would tend to increase social distancing efforts in order to limit the number of infections at any given time; see, e.g., ref. 26 for details.

It is very typical in control theory to assume quadratic control costs, because it has the advantage of being the most simple convex form. Using this for the social distancing cost ensures that $\kappa =R_{0}$ for a disease of negligible cost. This choice was also made by, e.g., Makris and Toxvaerd (12). One possible point of criticism for using a quadratic social distancing cost is that it stays finite for κ=0. This allows the population to unrealistically avoid all new infections for a finite cost per time unit. This issue could be addressed by instead using a functional form for the social distancing cost that diverges as κ→0 (5). However, the solutions studied here respect κ≥1 (Eq. **31**) so the lack of a divergence is not a concern here. If $t_{f}$ were chosen to be short enough so as to incentivize complete lockdown scenarios, a divergent control cost would instead have to be considered.

In other work, the social distancing term is often assumed to be proportional to $\psi_{s}$, e.g., refs. 5 and 78. This assumption corresponds to the following situation: a) Individuals know well in which compartment they currently reside. b) Individuals only social distance as long as they are susceptible, i.e., when they are still able to avoid infection. c) The behavior of infected individuals is constant. Either, infected individuals know that they cannot positively affect their own fate by continuing to social distance and thus do not do so, or, infected individuals show some constant social distancing behavior that is absorbed into the definition of $R_{0}$. d) Recovered individuals know that they can safely return to their pre-epidemic lifestyle and thus avoid excessive social distancing costs.

Instead, we chose to use a utility that does not include a $\psi_{s}$ factor in the social distancing term for simplicity. Indeed this simplification allows the derivation of the analytic solution that we identify. Briefly, this corresponds to the case in which individuals know that becoming infected would be very costly on average but are uncertain about which compartment they find themselves in. This is the case when many infections are asymptomatic and many individuals believe they are susceptible even when recovered (a similar assumption is made in ref. 19 who considers an asymptomatic stage of the disease that sometimes turns symptomatic). At the same time, we have assumed that the status of the epidemic is well known, so that individuals are able to make informed decisions while being unclear about their own status. A possible scenario is thus one in which costly disease symptoms are either delayed and/or rare, so that individuals would be uncertain about their infection status while wanting to avoid infection. In this scenario, we are assuming that there is unbiased testing of population infection levels such that the course of the disease remains predictable, such as testing sewage (88). Indeed, in the recent COVID-19 epidemic, a large fraction of individuals were asymptomatic, while costs arising from an infection, including death and long-COVID, could be very high. There are many other examples of diseases that fall into this class, e.g., HIV, Epstein–Barr, HPV, or Herpes; or diseases for which there are rare genetic susceptibilities (89). In all these cases, the individuals can remain unaware that they were infected indefinitely, while the costs of an infection can be very large. Since it can be costly to have recovered individuals social distance in perpetuity, a government would likely seek to institute large-scale testing to identify recovered individuals in the population so that they could return to a pre-epidemic activity level (29). We also must neglect that the utility/cost of social behavior for an individual would depend on how active other members of the population are, which may lead to the existence of multiple Nash equilibria (19).

One might ask what the practical difference between a utility with and a utility without the $\psi_{s}$ factor would be. In fact, we find that the behavior and epidemic trajectories obtained in this work are qualitatively comparable to what one would (numerically) obtain assuming that all infections are immediately symptomatic, e.g., see refs. 5 and 26. The main qualitative difference is that a utility in which social distancing is performed only by the susceptible compartment would predict that the most stringent social distancing is performed (at least slightly) after the epidemic has already peaked and when the susceptible compartment is already strongly reduced. Instead, we find here that the strongest reduction in social activity is expected exactly at the peak of the epidemic.

Our approach neglects any possible effects of government intervention which would for instance allow targeting utilitarian behavior. We have been unable to extend the analytic solution to the presence of state intervention so far. Nevertheless, the insights gained from the analytic solution may be useful in the design of government interventions.

### Restatement of the Problem as a Boundary Value Problem.

We use a standard Hamiltonian/Lagrangian approach (14, 86), which in optimal control theory is referred to as Pontryagin’s maximum principle (90), to calculate the optimal behavior of an individual κ in response to an exogenous behavior k and the corresponding course of the epidemic. This approach is an exact reformulation of a full variational analysis of the individual’s behavior and epidemic trajectory given an exogenous population behavior. For details, see for instance refs. 14 and 86 or Supplementary Information of ref. 26. It allows for exactly reformulating the optimization problem as a boundary value problem which is generated from an auxiliary function, the Hamiltonian. The Hamiltonian for the individual can be expressed by

[6] $$\begin{array}{cc} H= & \;u+v_{s}\frac{d\psi_{s}}{\mathrm{dt}}+v_{i}\frac{d\psi_{i}}{\mathrm{dt}}\\ = & \;u+v_{s}\left(-\kappa \psi_{s}i\right)+v_{i}\left(\kappa \psi_{s}i-\psi_{i}\right)\\ = & -\alpha \psi_{i}-\;{\left(\kappa -R_{0}\right)}^{2}-\left(v_{s}-v_{i}\right)\kappa \psi_{s}i-v_{i}\psi_{i}. \end{array}$$

The Lagrange fields $v_{s}\left(t\right)$ and $v_{i}\left(t\right)$ express the expected value of being in the corresponding compartment at that point in time (14). They enforce the constraint of the dynamics to Eq. **2**. Their equations of motion are

[7] $$\begin{array}{cc} \frac{d}{\mathrm{dt}}v_{s} & =-\frac{\partial H}{\partial \psi_{s}}=\left(v_{s}-v_{i}\right)\kappa i \end{array}$$

[8] $$\begin{array}{cc} \frac{d}{\mathrm{dt}}v_{i} & =-\frac{\partial H}{\partial \psi_{i}}=\alpha +v_{i}, \end{array}$$

with boundary conditions

[9] $$\begin{array}{c} v_{s}\left(t_{f}\right)=\frac{\partial U_{f}}{\partial \psi_{s,f}}=0,,\;v_{i}\left(t_{f}\right)=\frac{\partial U_{f}}{\partial \psi_{i,f}}=-\alpha . \end{array}$$

Given the exogenous course of the epidemic in the population, the individual can optimize their own utility by choosing the strategy that satisfies 0=∂H/∂κ. From this we obtain $\kappa =R_{0}-\frac{1}{2}\left(v_{s}-v_{i}\right)\psi_{s}i$. Assuming that the population consists of identical individuals, they all rationally seek to optimize their personal utility in the same way. Thus they all choose the same strategy and we can conclude that the average population behavior must be self-consistently given by k(t)=κ(t). Hence, this gives rise to a Nash equilibrium. Then, naturally also $s=\psi_{s}$ and $i=\psi_{i}$, and

[10] $$k=\kappa =R_{0}-\frac{1}{2}\left(v_{s}-v_{i}\right)si.$$

The variational approach as stated only yields conditions sufficient to identify extrema. To confirm that a solution is a maximum, $\frac{\partial^{2}H}{\partial \kappa^{2}}<0$ is required. Here, we find $\frac{\partial^{2}H}{\partial \kappa^{2}}=-2<0$.

## Analytic Solution

The Nash equilibrium k(t) optimizing the utility Eq. **4** is given by the solution of Eq. **1**, Eqs. **7**–**9**, in conjunction with the optimality condition Eq. **10**. From here, we calculate the analytic solution for this set of equations.

First, we work with the integrated fraction of infected cases up to time t, i.e., the fraction of recovered cases r, defined as

[11] $$r=\int_{0}^{t}i\left(t^{\prime }\right)dt^{\prime }+r_{0}$$

noting that i=dr/dt. Because r(t) is monotonic, it can be used as a rescaling of time. Thus we can rely on a one-to-one mapping between t and r for all time-dependent quantities, for instance between s(t) and s(r). This or similar reparameterizations of time are a common technique in the analysis of epidemic compartmental models, e.g., see refs. 80–82 and 91. What is innovative here is that we leverage this rescaling for a fully time-dependent population behavior k(t).

The second transformation involves defining dK/dt=ki, hence K obeys

[12] $$K\left(r\right)=\int_{r_{0}}^{r}k\left(r^{\prime }\right)dr^{\prime }.$$

Eq. **1** then lead to ds/dt=−sdK/dt which integrates to

[13] $$s=s_{0}e^{-K(r)}.$$

Using 1=s+i+r, we obtain directly

[14] $$i=1-r-s_{0}e^{-K(r)}.$$

Since i=dr/dt, we can integrate this equation to obtain

[15] $$F\left(r\right)\equiv \int_{r_{0}}^{r}\frac{dr^{\prime }}{1-r^{\prime }-s_{0}e^{-K(r^{\prime })}}=t\;\;\;\mathrm{hence}\;\;r=F^{-1}\left(t\right).$$

Here, we exploit that this mapping between r and t is valid for arbitrarily time-dependent population behavior k(t). We recall our assumption that $t_{f}$ is large enough that $i(t_{f})\to 0$. In this limit, Eq. **14** simplifies and the cumulative total of infections reaches its final value given by the root of

[16] $$r_{f}+s_{0}e^{-K(r_{f})}=1.$$

Eqs. **11**–**16** above hold irrespective of the form of the objective function.

In the following, we evaluate K, and hence s, for the particular choice and structure of Eq. **5**. Concerning the Lagrange fields, we can see directly from Eq. **8** that $v_{i}\left(t\right)=-\alpha$, whereas $v_{s}$ follows from Eq. **7**

[17] $$\frac{d}{\mathrm{dt}}v_{s}=\left(v_{s}+\alpha \right)\frac{d}{\mathrm{dt}}K.$$

Integrating we obtain

[18] $$v_{s}+\alpha =\mu e^{K}=\mu \frac{s_{0}}{s}.$$

with a constant μ. From the boundary condition $v_{s}\left(t_{f}\right)=0$ we can conclude

[19] $$v_{s}=-\alpha \frac{s-s_{f}}{s}$$

with $s_{f}=s\left(t_{f}\right)$. The quantity $v_{s}$ can be interpreted as the expected future infection cost for a susceptible individual, since the probability that they will still become infected is $(s-s_{f})/s$ at a cost −α. The optimal behavior is then given by Eq. **10**

[20] $$\begin{array}{cc} k & =R_{0}-\frac{\alpha s_{f}}{2}i. \end{array}$$

**Fig. 1.**
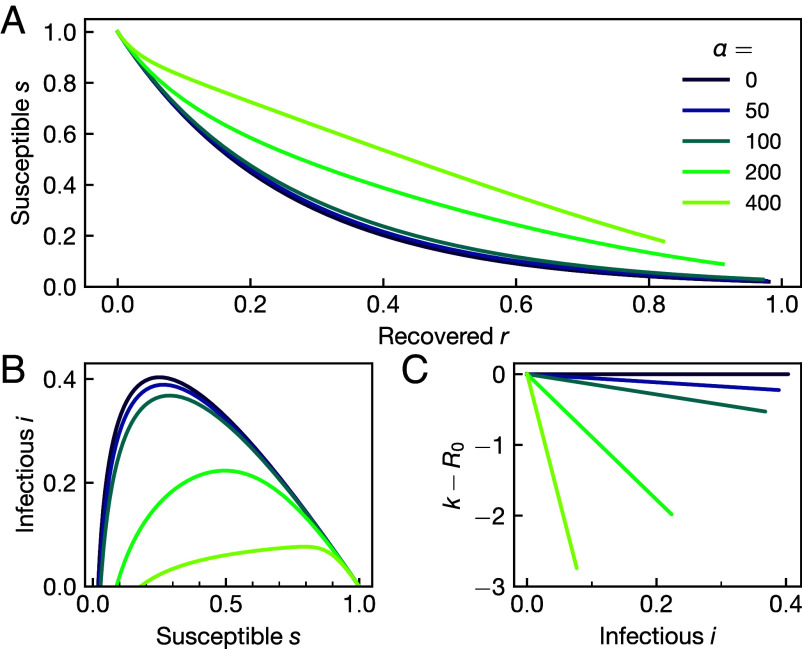
Direct plots of the analytic solution. (*A*) The analytic solution of the Nash equilibrium social distancing problem as obtained in Eq. **23** as a function of the recovered r for an exemplary range of infection costs α and $R_{0}=4$. Initial conditions here and in all following figures are set to $r_{0}=10^{-6}$ and $i_{0}=3\cdot 10^{-6}$. (*B*) The fraction of infectious i as a function of the susceptible s for the same range of α. (*C*) Deviation of the social distancing behavior k from the pre-epidemic default $R_{0}$ as a function of i, emphasizing their linear relationship as established in Eq. **20**.

This is tremendously simple: the equilibrium strength of social distancing $k-R_{0}$ is proportional to both the number of infectious cases and the cost of infection at any given time; see Fig. 1*C*.

With $s=s_{0}e^{-K(r)}$, we have

[21] $$\frac{\partial s}{\partial r}=-s\frac{\partial K}{\partial r}=-sk$$

and therefore, inserting Eq. **20** and i=1−r−s,

[22] $$\begin{array}{cc} \frac{\partial s}{\partial r} & =-s[R_{0}-\frac{\alpha s_{f}}{2}\left(1-r-s\right)]\\ & =-s[a+br+bs]. \end{array}$$

with $a=R_{0}-b$ and $b=\alpha s_{f}/2$. This has an analytic solution that satisfies $s\left(r\to r_{0}\right)\to s_{0}$

[23] $$s\left(r\right)=\frac{\exp\left[-\frac{1}{2}\left(r-r_{0}\right)\left(2a+b\left(r+r_{0}\right)\right)\right]}{\frac{1}{s_{0}}-\sqrt{\frac{\pi b}{2}}\exp\left[\frac{{\left(a+br_{0}\right)}^{2}}{2b}\right]\left(\;\text{Erf}\left[\frac{a+br_{0}}{\sqrt{2b}}\right]-\;\text{Erf}\left[\frac{a+br}{\sqrt{2b}}\right]\right)}$$

Using $r_{f}=1-s\left(r_{f}\right)$, we can self-consistently determine $r_{f}$ and thus obtain the solution. We show the result of Eq. **23** for a range of infection costs α in Fig. 1*A*. The analytic solution for the infectious compartment i(r)=1−r−s(r) can be plotted in a natural way on the *s*–*i* plane; see Fig. 1*B*.

**Fig. 2.**
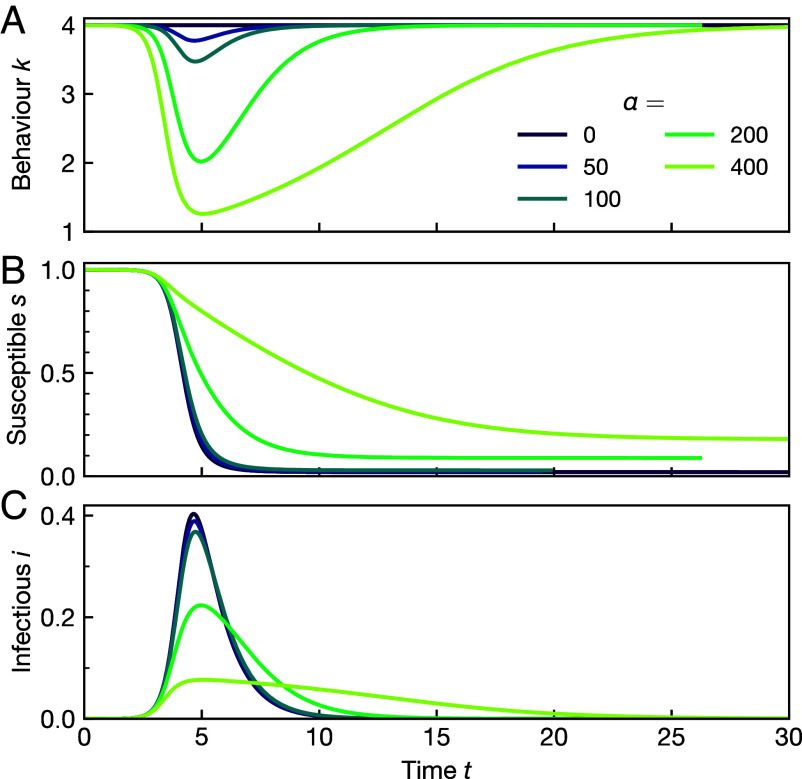
Analytic solution as a function of time. (*A*) Equilibrium social activity behavior of the population k(t) and corresponding dynamics of the disease (*B*) s and (*C*) i for an exemplary range of infection costs α and $R_{0}=4$. Since infections incur a cost, the equilibrium behavior seeks to avoid excessive infections by self-organized social distancing. The higher the cost, the more reduced social activity k becomes.

In our approach, time is parameterized as Eq. **15**, which can easily be evaluated numerically. The analytic solution can then be plotted in the typical way, Fig. 2.

## Results

The central result of this work is that we have been able to obtain a full analytic solution to the epidemic dynamics in Eq. **23** and that it arises from a simple expression for the fully time-dependent social distancing behavior that can be expected under rational decision making, Eq. **20**. This equation formalizes an intuitively reasonable result: the higher the infection cost α, the stronger is the incentive to reduce social activity and hence k, see Fig. 2. The stronger the reduction in k, the more slowly the epidemic progresses, the lower the peak infection levels are, and the lower the total number of cases $1-s_{f}$ becomes.

In what follows, we analyze the epidemic using two key quantities, the excess cases ε and the peak of the epidemic max(i). For $t_{f}\to \infty$, herd immunity is always reached. The final number of susceptibles then always satisfies $s_{f}\leq 1/R_{0}$, with $1/R_{0}$ the minimum number of cases for which herd immunity is guaranteed. The cases in excess of this threshold are defined as

[24] $$\varepsilon =1/R_{0}-s_{f}.$$

We will calculate ε and max(i) in two limiting cases: 1) The Nonbehavioral limit in which there is no perceived infection cost α=0. In this case, there is no reason to modify one’s behavior, $k=R_{0}$; see purple lines in Figs. 1 and 2. 2) The high-infection-cost asymptote in which infection costs are very high, $\alpha /R_{0}^{2}\gg 1$. By matching these solutions, we will obtain crossover costs between these scaling results.

### Nonbehavioral Limit.

For this edge case only, the analytic solution was known previously (1, 80–83). We recover it in our notation as follows. Since α=0, Eq. **22** is solved by

[25] $$s\left(r\right)=s_{0}e^{-R_{0}\left(r-r_{0}\right)}.$$

Its limit $s_{f}=e^{-R_{0}\left(1-s_{f}-r_{0}\right)}$ yields

[26] $$s_{f}=-W\left(-s_{0}R_{0}e^{R_{0}\left(r_{0}-1\right)}\right)/R_{0}$$

with W the principal branch of the Lambert W function, which is also called the product logarithm and is defined as the inverse of the function $we^{w}$ (92). Hence,

[27] $$\varepsilon =\left(1+W\left(-s_{0}R_{0}e^{R_{0}\left(r_{0}-1\right)}\right)\right)/R_{0}.$$

The peak of the epidemic $\hat{i}=\text{max}\left(i\right)=i\left(\hat{t}\right)$ occurs at the time $\hat{t}$ for which di/dt=0 and thus $s\left(\hat{t}\right)=1/R_{0}$; see Eq. **1**. Inserting this and r=1−s−i into Eq. **25**, we obtain

[28] $$\hat{i}=\;\text{max}\left(i\right)=1-r_{0}-\left(1+\text{ln}\left(s_{0}R_{0}\right)\right)/R_{0}.$$

### High-Infection-Cost Asymptote.

The final number of cases $s_{f}$ can be calculated in the limit of large $\alpha \gg R_{0}^{2}$, where $s_{f}=1/R_{0}-\varepsilon$ with ε small and assuming that $s_{0}>1/R_{0}$. We obtain

**Fig. 3.**
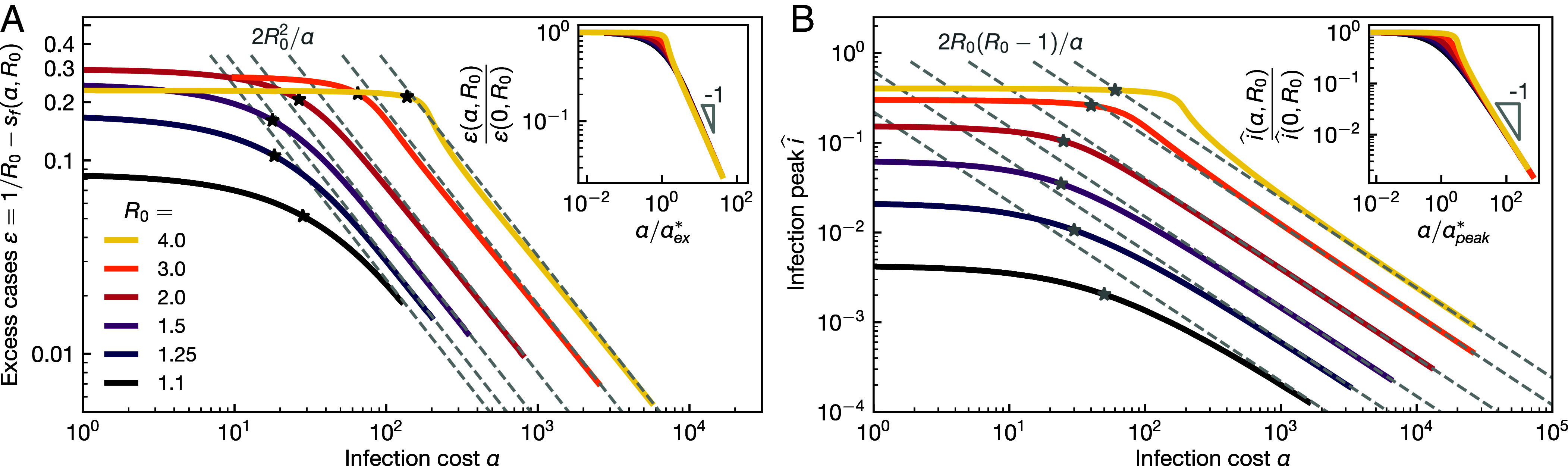
Scaling. (*A*) Excess cases $\varepsilon (\alpha ,R_{0})$ vs. infection cost α for a range of basic reproduction numbers $R_{0}$. The high infection cost asymptotes, see Eq. **29**, are shown as dashed lines and the crossover costs $\alpha_{\mathrm{ex}}^{⋆}$, see Eq. **32**, as black stars. Inset: The data collapse onto the low and high infection cost asymptotes by rescaling the cost α with the crossover cost $\alpha_{\mathrm{ex}}^{⋆}$, see Eq. **32**, while rescaling $\varepsilon (\alpha ,R_{0})$ with its nonbehavioral limit, see Eq. **27**. (*B*) The infection peak $\hat{i}$ vs. α for a range of $R_{0}$. The high infection cost asymptotes, see Eq. **30**, are shown as dashed lines and the crossover costs $\alpha_{\mathrm{peak}}^{⋆}$, see Eq. **33**, as gray stars. *Inset*: The data collapse onto the low and high infection cost asymptotes by rescaling the cost α with the crossover cost $\alpha_{\mathrm{peak}}^{⋆}$, Eq. **33**, while rescaling the peak height with its nonbehavioral limit, see Eq. **28**.

[29] $$\varepsilon =2R_{0}^{2}/\alpha$$

from an expansion of Eq. **23** in both 1/α and ε small and by matching order by order. This result describes the full solution well at high α; see Fig. 3*A*. For the infection peak, we obtain in the same limit, see *Materials and Methods*,

[30] $$\hat{i}=\;\text{max}\left(i\right)=2R_{0}\left(R_{0}-1\right)/\alpha ,$$

which also fits the full solution well at high α; see Fig. 3*B*. In the limit α→∞ we combine Eqs. **20**, **29**, and **30** and see that asymptotically

[31] $$\begin{array}{cc} k & =R_{0}-\frac{\alpha s_{f}}{2}i\geq R_{0}-\left(1-2R_{0}^{3}/\alpha \right)\left(R_{0}-1\right)\geq 1 \end{array}$$

### Scaling and Phase Diagram.

It can be illuminating to calculate the approximate infection cost at which self-organized behavior starts to play a significant role. For this purpose, we identify the costs at which low and high infection cost asymptotes meet. We find two such crossover costs, one for excess cases, $\alpha_{\mathrm{ex}}^{⋆}$, and one for the infection peak, $\alpha_{\mathrm{peak}}^{⋆}$, as follows. Observing in Fig. 3*A* that the excess cases are roughly constant at low α and therefore well described by the nonbehavioral limit, we obtain the crossover cost $\alpha_{\mathrm{ex}}^{⋆}$ at which the nonbehavioral and high infection cost asymptotes of Eqs. **27** and **29**, respectively, match

[32] $$\alpha_{\mathrm{ex}}^{⋆}=2R_{0}^{3}/\left(1+(W\left(-s_{0}R_{0}e^{R_{0}\left(r_{0}-1\right)}\right)\right).$$

For the infection peak, we similarly obtain a crossover cost $\alpha_{\mathrm{peak}}^{⋆}$ from matching Eqs. **28** and **30**

[33] $$\alpha_{\mathrm{peak}}^{⋆}=2R_{0}^{2}\left(R_{0}-1\right)/\left(R_{0}\left(1-r_{0}\right)-1-\text{ln}\left(s_{0}R_{0}\right)\right).$$

These crossover values and the nonbehavioral limits for ε and max(i) can be used to achieve complete collapse of ε and max(i) onto master curves; see Fig. 3 *B* and *D*, respectively.

**Fig. 4.**
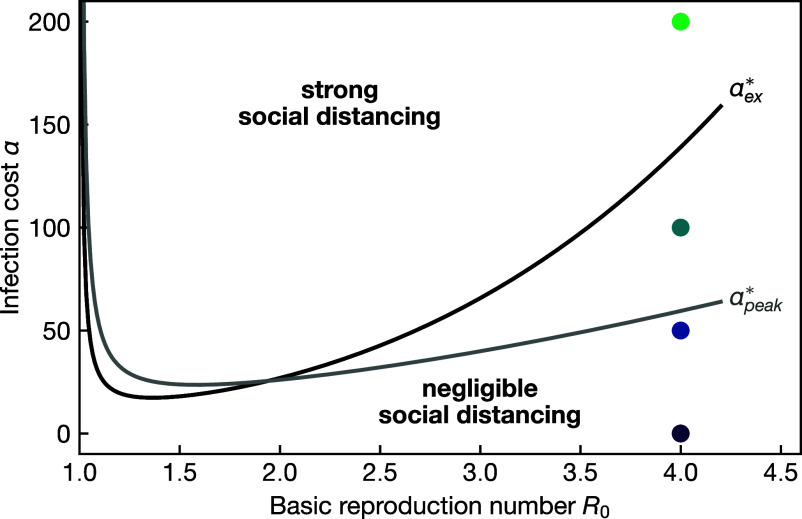
Behavioral response. Characterization of the Nash equilibrium response in the $R_{0}$—α parameter space. On the high $R_{0}$—low-α side of the line, the behavior is well represented by the nonbehavioral limit, in which it is not rational to significantly modify one’s behavior. On the low $R_{0}$—high infection cost side, it is rational to strongly modify one’s behavior. The lines describing the crossover are given by the critical costs $\alpha_{\mathrm{ex}}^{⋆}$ for the transition in the excess cases, see Eq. **32**, and/or $\alpha_{\mathrm{peak}}^{⋆}$ for the transition in the infection peak, see Eq. **33**. The parameter values used for some of the curves in Figs. 1 and 2 are marked by analogously colored dots.

Both crossover values, $\alpha_{\mathrm{peak}}^{⋆}$ and $\alpha_{\mathrm{ex}}^{⋆}$, determine different aspects of the “phase diagram” of social distancing; see Fig. 4. For $R_{0}=4$, $\alpha_{\mathrm{peak}}^{⋆}\approx 59$ and $\alpha_{\mathrm{ex}}^{⋆}\approx 139$. The crossover $\alpha_{\mathrm{peak}}^{⋆}$ for the infection peak describes a behavioral transition in the most intuitive signal of an epidemic. The infection peak also corresponds to the most restrictive value of social distancing; see Eq. **20**. For $\alpha <\alpha_{\mathrm{peak}}^{⋆}$ social distancing is extremely weak; see, e.g., for α=50 in Fig. 2*A*. Social distancing is ultimately aimed at reducing excess cases. For $\alpha_{\mathrm{peak}}^{⋆}\leq \alpha \leq \alpha_{\mathrm{ex}}^{⋆}$, there is social distancing, but still only on a relatively short time frame; see the data for α=100 in Fig. 2*A*. It starts to visibly affect the peak of the epidemic but not its duration, Fig. 2*C*, and has a very limited effect on the total of cases, Fig. 2*B*. This can be viewed as the consequence of the weak relationship between the drop in infectivity and excess cases. Only for $\alpha >\alpha_{\mathrm{ex}}^{⋆}$ is there considerable social distancing for an extended time, which then achieves a significant reduction in excess cases.

### Utility.

The utility, Eq. **4**, evaluated at the equilibrium behavior can be directly calculated using the analytic solution

**Fig. 5.**
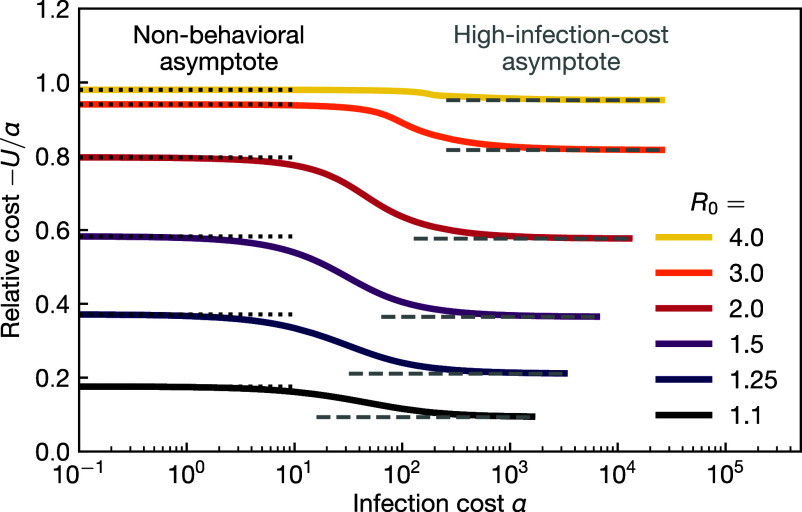
Cost of the epidemic. Total epidemic cost relative to the cost of an infection, −U/α, as a function of infection cost α under equilibrium social distancing. The corresponding nonbehavioral, Eq. **35**, and high-infection-cost asymptotes, Eq. **36**, are indicated by dotted and dashed lines, respectively.

[34] $$\begin{array}{cc} U & =-\alpha \left[r_{f}-r_{0}+\frac{s_{f}}{2}\left(R_{0}\left(r_{f}-r_{0}\right)+\text{ln}\left(s_{f}/s_{0}\right)\right)\right] \end{array}$$

noting that $s_{f}$ and $r_{f}=1-s_{f}$ depend on α and $R_{0}$. The total infection cost is given by $-\alpha (r_{f}-r_{0})$ with the remainder being the total social distancing cost. Especially for intermediary $R_{0}$ and high infection costs α, equilibrium behavior strongly reduces the total epidemic cost; see Fig. 5.

Again, we investigate the two limiting cases: for low α, we obtain with Eq. **26** the nonbehavioral result

[35] $$\begin{array}{cc} U & =-\alpha \left[1+\frac{1}{R_{0}}W\left(-s_{0}R_{0}e^{R_{0}\left(r_{0}-1\right)}\right)-r_{0}\right] \end{array}$$

The difference between Eqs. **34** and **35** precisely quantifies the utility gained by self-organized social distancing. This gain is particularly high for high infection cost. In that limit, $\alpha /R_{0}^{2}\gg 1$, the utility is asymptotically given by, with $s_{f}=\frac{1}{R_{0}}-\varepsilon =\frac{1}{R_{0}}-\frac{2R_{0}^{2}}{\alpha }\approx 1/R_{0}$

[36] $$\begin{array}{cc} U & =-\alpha \left[\frac{3}{2}\left(1-r_{0}\right)-\frac{3+\ln(R_{0}s_{0})}{2R_{0}}\right] \end{array}$$

## Discussion and Conclusion

In summary, we have identified an analytic solution for the fully time-dependent Nash equilibrium behavior for social distancing during an epidemic. We leveraged this solution to obtain the following four key results.

First, a simple expression for the strength of rational social distancing that is proportional to both the current number of cases and the cost of infection, Eq. **20**. This result provides a rigorous justification for models that assume a reduction of behavioral activity with prevalence (2, 4, 6, 17), e.g., in models developed for tackling the HIV epidemic (93, 94). This justification is especially strong in cases where our choice for the utility is well aligned with the characteristics of the disease: infections that are initially asymptomatic with delayed symptoms at high cost. This is exactly the profile of HIV and similar diseases.

Second, scaling results for the total number of cases, Eq. **29**, and the infection peak, Eq. **30** which only depend on the basic reproduction number and the cost of contracting the disease. Such scaling results could have been previously accessible for behavioral models using numerical approaches, e.g., refs. 5, 12, 19, and 26. Qualitatively, the reduction of the peak and excess cases with infection cost was known, e.g., see ref. 87, but an explicit scaling with disease and utility parameters has not been reported to our knowledge. Earlier analytic approaches (1, 80–83) did not allow for fully time-varying behavior, in contrast to the current work.

Third, characteristic infection costs, Eqs. **32** and **33**, that divide regimes of strong and weak social distancing and depend only on the basic reproduction number of the disease. Similar results are known numerically (5).

Finally, a closed form expression for the value of the utility, Eq. **34**, which allows quantifying the expected increase of utility due to self-organized social distancing. Similar results are known numerically (5).

These four results represent a remarkable simplification of a complex optimization problem. The advantage of having an analytic solution is always that it provides a deep and intuitive understanding; here, of self-organized behavior in epidemics. Of course, it is always possible to extend the complexity of the model, e.g., beyond a vanilla SIR model. However, this will almost surely result in an approach that must rely on numerical techniques and thus can give approximate solutions while compromising the deep overarching understanding. For other choices of the utility, for instance corresponding to perfectly symptomatic infections or assuming heterogenous utilities, the exact functional form of these results would likely be different. Nevertheless, the results reported here may prove useful as analytic approximations.

We believe our work to be useful to policy makers because it yields a simple, albeit idealized, classification of the impact of self-organized social distancing during epidemics and thus can serve as guide for policy. Given the basic reproduction number of a given disease $R_{0}$ and its estimated cost of infection α, we show that one can either expect negligible social distancing from the population, when the infection cost is below a characteristic cost, or substantial social distancing when it is above.

There is an ongoing debate about the degree to which behavior of individuals is truly rational, as we (and others) assume. In this context, our most significant result is that the rational decision making process seems to be intuitively accessible to most members of the population: rational social distancing is proportional to the infection cost and to the current number of cases. It is remarkable that the rational response we derive can be condensed into such a simple heuristic, understandable to a typical member of the population. While it may indeed be a challenge for such individuals to derive our results for themselves, a policymaker could communicate this simple heuristic, to be adopted by the population in order to assist them in targeting truly rational behavior. It is not unrealistic to expect this advice to influence the population decision making, especially given that it can be shown to be in each individual’s self-interest. In this sense, the present work may itself help to “bootstrap” such rational behavior.

While rational behavior is not the mathematically optimal solution that maximizes utility, as would be accessible under arbitrarily precise government control, it is relatively close to it. Rational behavior also has the advantage of being stable, in the sense that it suppresses the detrimental behavior of freeloaders, who are worse off if they deviate from the Nash equilibrium behavior. The fact that rational behavior is so desirable means that new tools that enable policymakers to help individuals target rational behavior, like the ones we provide here, may be extremely valuable.

The analytic solution derived here can serve as a starting point for semiexact or perturbative solutions of more complex disease models and utilities, for instance with heterogenous population structure.

## Materials and Methods

### Vaccination Salvage Term.

A perfect vaccine applied to the whole population at time $t_{f}$ corresponds to immediately moving the susceptible fraction of the population into the recovered compartment, $s(t>t_{f})=0$ and $\psi_{s}\left(t>t_{f}\right)=0$. Eq. **1** reduce to

[37] $$\begin{array}{cc} \frac{d}{\mathrm{dt}}i & =-i. \end{array}$$

The remaining infectious recover exponentially, with $i\left(t_{f}\right)=i_{f}$,

[38] $$i\left(t>t_{f}\right)=i_{f}\text{exp}\left[-\left(t-t_{f}\right)\right].$$

Analogously for the individual probabilities, with $\psi_{i}\left(t_{f}\right)=\psi_{i,f}$,

[39] $$\psi_{i}\left(t>t_{f}\right)=\psi_{i,f}\text{exp}\left[-\left(t-t_{f}\right)\right].$$

Since there are no new infections, the population selects pre-epidemic behavior, $\kappa \left(t>t_{f}\right)=R_{0}$. The contribution to the utility $U_{f}$ that arises from the recovery process after $t_{f}$ can be written in analogy to Eqs. **4** and **5**

$$\begin{array}{cc} U_{f} & =\int_{t_{f}}^{\infty }-\alpha \;\psi_{i}\left(t\right)-{\left(\kappa \left(t\right)-R_{0}\right)}^{2}dt. \end{array}$$

This can be integrated to yield

[40] $$U_{f}=-\alpha \;\psi_{i,f}.$$

### High-Infection Cost Asymptote for the Infection Peak Height.

In the large α limit, $\alpha /R_{0}^{2}\gg 1$, we have $s_{f}\approx 1/R_{0}$ from Eq. **24**, hence

[41] $$b=\alpha s_{f}/2\approx \alpha /\left(2R_{0}\right)$$

large according to $b\gg R_{0}$. The infection peak di/dt=0 occurs at $\hat{i}=\text{max}\left(i\right)$, where Eq. **1** yields 0=ks−1. Using Eq. **20**, we have

[42] $$1=ks=s\left(R_{0}-\hat{i}b\right)\Rightarrow s=1/\left(R_{0}-\hat{i}b\right)$$

with the sum rule,

[43] $$\begin{array}{c} \hat{i}=1-r-s=1-r-1/\left(R_{0}-\hat{i}b\right)\\ \Rightarrow \left(R_{0}-\hat{i}b\right)\hat{i}=\left(1-r\right)\left(R_{0}-\hat{i}b\right)-1. \end{array}$$

This yields a quadratic equation for $\hat{i}$ with physical root

[44] $$\begin{array}{cc} \hat{i} & =\frac{R_{0}+b\left(1-r\right)-\sqrt{{\left(R_{0}+b\left(1-r\right)\right)}^{2}+4b\left(1-\left(1-r\right)R_{0}\right)}}{2b}\\ & \;\approx \frac{\left(1-r\right)}{2}\left[\frac{4b(\left(1-r\right)R_{0}-1)}{2{\left(R_{0}+b\left(1-r\right)\right)}^{2}}\right]. \end{array}$$

For large α the infection peak occurs early in the epidemic, when r=1−s−i≪1, e.g., see Fig. 2 for α=400. Using Eq. **41** and recalling also that $b\gg R_{0}$ we find

[45] $$\begin{array}{cc} \hat{i} & \approx 2R_{0}\left(R_{0}-1\right)/\alpha . \end{array}$$

## Data Availability

There are no data underlying this work.
